# Targeting Tularemia: Clinical, Laboratory, and Treatment Outcomes From an 11-year Retrospective Observational Cohort in Northern Sweden

**DOI:** 10.1093/cid/ciae098

**Published:** 2024-02-23

**Authors:** Martin Plymoth, Robert Lundqvist, Anders Nystedt, Anders Sjöstedt, Tomas N Gustafsson

**Affiliations:** Department of Clinical Microbiology, Sunderby Research Unit, Umeå University, Umeå, Sweden; Department of Infectious Diseases, Westmead Hospital, Sydney, New South Wales, Australia; Department of Public Health and Clinical Medicine, Sunderby Research Unit, Umeå University, Umeå, Sweden; Department of Communicable Disease Control, County Council of Norrbotten, Luleå, Sweden; Department of Clinical Microbiology, Umeå University, Umeå, Sweden; Department of Clinical Microbiology, Sunderby Research Unit, Umeå University, Umeå, Sweden

**Keywords:** *Francisella tularensis*, doxycycline, ciprofloxacin, treatment, outcome

## Abstract

**Background:**

Tularemia is an important reemerging disease with a multimodal transmission pattern. Treatment outcomes of current recommended antibiotic regimens (including ciprofloxacin and doxycycline) remain unclear. In this retrospective cohort study, we report clinical, laboratory, geographical, and treatment outcomes of laboratory-confirmed tularemia cases over an 11-year period in Northern Sweden.

**Methods:**

Data from reported tularemia cases (aged >10 years at time of study) in Norrbotten county between 2011 and 2021 were collected through review of electronic medical records and participant questionnaires; 415 of 784 accepted participation (52.9%). Of these, 327 were laboratory-confirmed cases (serology and/or polymerase chain reaction). A multivariable logistic regression model was used to investigate variables associated with retreatment.

**Results:**

Median age of participants was 54 years (interquartile range [IQR], 41.5–65) and 49.2% were female. Although ulceroglandular tularemia was the predominant form (n = 215, 65.7%), there were several cases of pulmonary tularemia (n = 40; 12.2%). Inflammatory markers were largely nonspecific, with monocytosis frequently observed (n = 36/75; 48%). Tularemia was often misdiagnosed on presentation (n = 158, 48.3%), with 65 (19.9%) receiving initial inappropriate antibiotics and 102 (31.2%) retreated. Persistent lymphadenopathy was infrequent (n = 22, 6.7%), with 10 undergoing surgical interventions. In multivariable analysis of variables associated with retreatment, we highlight differences in time until receiving appropriate antibiotics (8 [IQR, 3.25–20.75] vs 7 [IQR, 4–11.25] days; adjusted *P* = .076), and doxycycline-based treatment regimen (vs ciprofloxacin; adjusted *P* = .084), although this was not significant after correction for multiple comparisons.

**Conclusions:**

We comprehensively summarize clinical, laboratory, and treatment outcomes of type B tularemia. Targeting tularemia requires clinical awareness, early diagnosis, and timely commencement of treatment for an appropriate duration.

## Teaser

Treating tularemia is challenging because of a multitude of clinical presentations, and optimal treatment regimens remain unknown. We analyzed the outcomes of 327 laboratory-confirmed tularemia cases in Sweden and highlight the need for early diagnosis and initiating appropriate antibiotic treatment.

Tularemia is a reemerging zoonotic disease present in most countries in the Northern Hemisphere [1, 2]. Outbreaks of tularemia caused by the bacterium *Francisella tularensis* subsp. *holarctica* (“type B”) have occurred at irregular intervals in the northern parts of Sweden since first described in the country in 1931 [3, 4]. Although there is notable annual fluctuation, there has been an increasing trend in the number of cases seen across Sweden and the entire European Union over the latest 5-year period (2017–2021) [5, 6]. Increasing frequency of outbreaks in middle and southern parts of Sweden suggests tularemia is a reemerging infectious disease complexly linked with climate change [6–14].

Zoonotic, waterborne, airborne, and vector (primarily mosquito)-borne outbreaks of the disease have important health-related and socioeconomic burden on individuals and society through prolonged illness, absence from work, and healthcare costs [6].

Manifestations of the disease vary depending on inoculation dose, type of exposure (oral, ocular, pulmonary, cutaneous), and geographical location (type A in North America vs type B globally), with the latter causing debilitating symptoms, but rarely resulting in lethalities [4, 15, 16].

Recommended treatment regimens for tularemia include aminoglycosides (streptomycin and gentamicin), quinolones (ciprofloxacin), and tetracyclines (doxycycline), but knowledge of their in vivo effectiveness remains scarce [6, 17–20]. Based on limited available evidence, the World Health Organization recommends treatment with ciprofloxacin 500 mg twice daily for 10 days, or doxycycline 100 mg twice daily for 14 days for milder disease in adults, but comparisons of treatment outcomes are limited to small case series [18, 21, 22].

In this study, we comprehensively investigated clinical, microbiological, laboratory, and treatment outcomes in a cohort of tularemia cases in northern Sweden, which includes the second (2019) and third (2015) largest outbreaks recorded in Sweden.

## METHODS

### Study Setting

This study was performed in Norrbotten County, the largest (98 245 km^2^) and northernmost county in Sweden, with a sparse population of approximately 250 000 [23].

### Study Design and Procedure

This is a retrospective cohort study involving electronic medical record (EMR) review of tularemia cases in Norrbotten in combination with a targeted questionnaire to further explore clinical details. Cases were identified via the National Infection Control database (SmiNet). Inclusion criteria were: (1) diagnosis 2011–2021; (2) alive at time of study; (3) available contact details; and (4) age ≥10 years at time of study consent. Exclusion criteria were: (1) nonresponse/declined study participation; (2) unavailable EMR documentation; (3) improbable clinical diagnosis; or (4) not meeting World Health Organization presumptive or confirmed tularemia case definition [18].

Eligible participants were sent a letter containing a questionnaire, consent form, study information sheet, and web link to a secure survey platform (EvaSysV8.1, Region Norrbotten). Nonrespondents were sent notifying mobile text messages and a second paper questionnaire.

### Serology and Microbiology

Serological analysis was performed at the regional clinical microbiology laboratory at Sunderby hospital, Luleå, Sweden, using an immunochromatographic rapid test (VIRapid, Vircell, Granada, Spain) [24]; and the national reference laboratory for tularemia at Umeå University hospital, Umeå, Sweden, using a validated in-house enzyme-linked immunosorbent assay, together with polymerase chain reaction (PCR) and cultures [25].

### Statistical Analysis

Data were collected from the regional EMR (VAS 49.0, Region Norrbotten), including documentation from general practitioners (GPs), hospitals, and specialist clinics. Integrated electronic prescriptions and microbiological data were reviewed. Geographical information of reported site of infection was mapped using Epi Info 7.2.5 (Centre for Disease Control). Statistical analysis was performed in SPSS 28.0 (IBM). Statistical methods included logistic regression (categorical variables) and Mann-Whitney *U* test (continuous and ordinal variables) for univariate analysis; a multivariable logistic regression model using a stepwise backwards elimination protocol (Supplementary Table 1) [26]. Holm-Šídák correction was applied, with a corrected *P* value <.05 considered significant.

### Ethics

Ethics committee approval was obtained from The Swedish Ethical Review Authority (Dnr 2020-04411, 2021-02953) and Norrbotten County Research Council (Dnr 01690-2020). All study participants received written age-appropriate information about the study and provided written informed consent. Additionally, parental consent was obtained for individuals aged <15 years.

## RESULTS

### Study Participants

**Figure 1.**
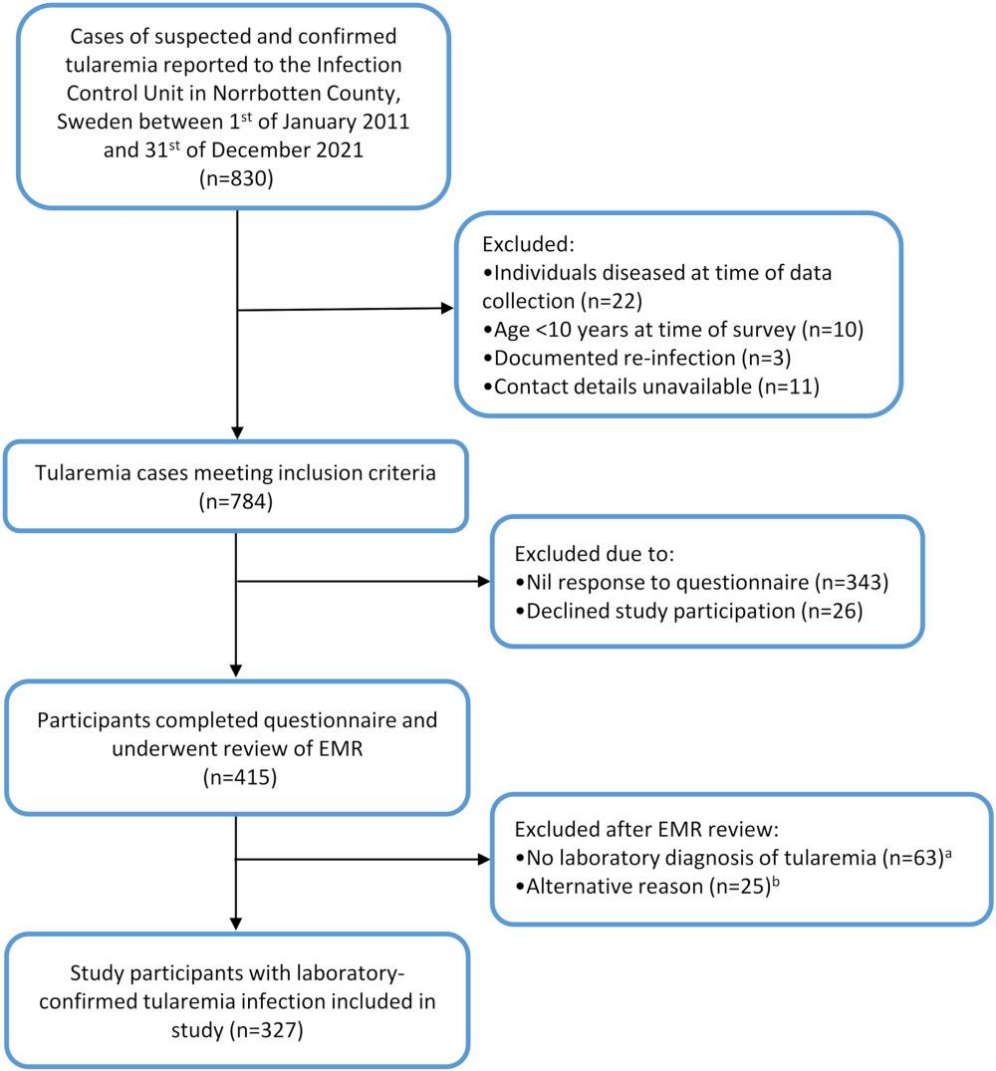
Study method and inclusion of participants. EMR, electronical medical record. ^a^Lack of electronical medical record documentation (n = 15); confirmed false-positive tularemia tests (n = 2; ×1 false-positive rapid test, ×1 false-positive PCR); misdiagnosis (later confirmed as mycoplasma infection via PCR; n = 1); incidental positive serology without evident clinical symptoms of tularemia infection (n = 5); age <10 y at time of survey (n = 2). ^b^Clinical diagnosis of tularemia without a positive tularemia serology, culture, and/or PCR and were therefore excluded from further analysis (of these, 15 had a single negative serology result and 1 had a negative PCR result). PCR, polymerase chain reaction.

In total, 830 cases of suspected or confirmed tularemia were reported to the Department of Communicable Disease Control, Norrbotten County, Sweden, between 1 January 2011 and 31 December 2021 (incidence, 30.1 per 100 000 per year). Three cases of tularemia reinfection were reported, all without laboratory confirmation. Overall, there were 441 questionnaire respondents between 30 December 2020 and 16 October 2022 (56.3% response rate), with 26 declining study participation. A further 88 of 415 were excluded after EMR review (Figure 1).

Study participants had a median age of 54 years at time of diagnosis (interquartile range [IQR], 46–65) and equal gender distribution (Table 1). Compared with all reported adult cases (aged ≥18 years), adult study participants (respondents) were older (median, 55 [IQR, 45–65] vs 53 [IQR, 40–65] years; *P* = .034) and less likely to be male (50.7% vs 57.8%; *P* = .049), but did not vary with regard to time since diagnosis (median, 6 [IQR, 6–9] vs 6 [IQR, 6–9] years; *P* = .088).

**Table 1.** Demographic, Microbiological, Clinical, and Laboratory Manifestations of Laboratory-Diagnosed Tularemia Cases and Associated Abnormal Values (n = 327)

	Laboratory-Diagnosed Tularemia Cases (n = 327)	Values Outside Laboratory Reference Range (%)
Age (y)	54 (IQR, 41.5–65)	
10–19	24 (7.3%)	
20–39	50 (15.3%)	
40–59	128 (39.1%)	
60–79	120 (36.7%)	
≥80	5 (1.5%)	
Gender		
Male	166 (50.7%)	
Female	161 (49.2%)	
Immunosuppression		
Mildly immunocompromised^a^	3 (0.9%)	
Severely immunocompromised^b^	6 (1.8%)	
Diabetes mellitus	1 (type 1, 0.3%); 17 (type 2, 5.2%)	
Pregnant at time of diagnosis	0 (0%)	
Clinical manifestations		
Ulceroglandular	215 (65.7%)	
Glandular	34 (10.4%)	
Pulmonary	40 (12.2%)	
Typhoidal	24 (7.3%)	
Oculoglandular	1 (0.3%)	
Oropharyngeal	0 (0%)	
Undifferentiated^c^	23 (7.0%)	
Microbiological diagnosis		
Positive *F. tularensis* serology titer (n = 311)	298 (95.8%)	
Seroconversion or significant rise in titer	114 (36.7%)	
PCR positive (n = 46)	44 (95.6%)	
Culture grown *F. tularensis*	18 (39.1%)	
Laboratory findings		
CRP (mg/L; n = 288)	60 (30–108)	
Leukocytes (×10^6^ cells/mL; n = 198)^d^	7.6 (6.2–9.3)	>8.8: 58 (29.3%)
Neutrophils (×10^6^ cells/mL; n = 71)	5.1 (3.6–6.2)	>6.1: 21 (29.6%)
Lymphocytes c (×10^6^ cells/mL; n = 76)	1.6 (1.1–2.3)	<1.1: 16 (21%) > 3.5: 3 (3.9%)
Monocytes c (×10^6^ cells/mL; n = 75)	0.7 (0.5–1.0)	>0.8: 36 (48.0%)
Eosinophils (×10^6^ cells/mL; n = 74)	0.09 (0.080–0.090)	>0.5: 0 (0%)
Basophils (×10^6^ cells/mL; n = 73)	0.03 (0.010–0.050)	>0.1: 2 (2.7%)
Hemoglobin (g/dL; n = 212)	137 (128–146)	<120 (female) or <130 (male): 40 (18.9%)
Platelets (×10^6^/mL; n = 204)	216 (168–300)	<150: 28 (13.7%) > 300: 50 (24.5%)
Procalcitonin (ng/mL; n = 17)	0.21 (0.13–0.34)	>0.1: 15 (88.2%) > 0.25: 6 (35.3%)
Urine analysis (n = 33)		
Hematuria	18 (54.5%)	Trace: 4 (12.1%) 25: 4 (12.1%) 80: 8 (24.2%) 200: 2 (6%)
Proteinuria	17 (51.5%)	Trace: 1 (3%) 0.3 (+): 10 (30.3%) 1.0 (++) 6 (18.2%)
Pyuria	7 (21.2%)	15: 5 (15.2%) 70: 1 500: 1

Abbreviations: CRP, C-reactive peptide; *F. tularensis*, *Francisella tularensis*; PCR, polymerase chain reaction.

^a^Low-dose corticosteroids (equivalent to <20 mg prednisolone; n = 2); methotrexate (≤0.4 mg/kg/week; n = 1).

^b^Infliximab and methotrexate (n = 2); Etanercept 50 mg and methotrexate 15 mg weekly (n = 1); golimumab monthly and methotrexate 15 mg weekly (n = 1); cyclosporine (n = 1); etanercept and sulfasalazine 500 mg 4 times per day (n = 1).

^c^Twelve participants (3.7%) had ulcers without documented lymphadenopathy and a further 11 (3.4%) had nonspecific mild symptoms.

^d^Participants aged <18 years were excluded because of alternative cutoff values.

**Figure 2.**
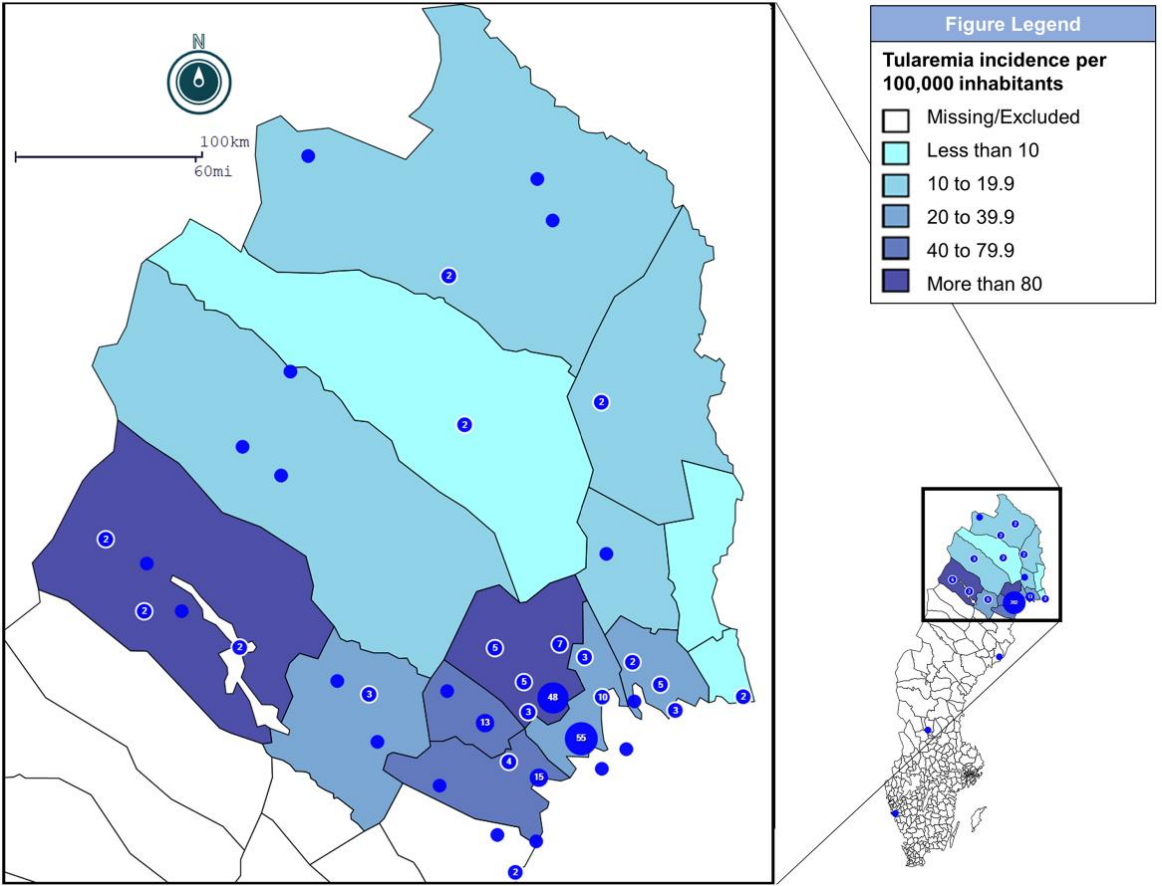
Geographical distribution and epidemiological incidence of tularemia cases at municipality level in Norrbotten County over 11 y between 2011 and 2021 (n = 243). Incidence calculated using total number of reported cases (N = 830). Blue dots indicate self-reported location of exposure/illness by individual cases. Number in blue dots indicate cumulative number of cases in area.

Geographical distribution (Figure 2) showed predominance of tularemia cases in coastal municipalities, as well as high incidence (>80 per 100 000 inhabitants) in certain highland areas popular among tourists, with a high density of lakes and waterways.

### Microbiological Analyses

Of 327 laboratory-confirmed cases, 298/311 (95.8%) had at least 1 positive serology, whereas 13/311 had 1 negative serology taken early on, combined with positive PCR and/or culture (Table 1). Overall, 114 (36.7%) had a documented seroconversion or significant rise in antibody titer [25]. *F. tularensis* PCR was positive in 43/43 (100%) samples from peripheral sites, 1/1 (100%) transbronchial needle aspirates, and 0/2 (0%) cerebrospinal fluid samples. *F. tularensis* (n = 18) was cultured from peripheral swabs (n = 13), blood (n = 3), pleural fluid (n = 1), and lymph node biopsy (n = 1).

### Clinical Manifestations

Visible skin ulceration and regional lymphadenopathy were seen among 215 (65.7%) participants (ulceroglandular tularemia), whereas 34 (10.4%) had isolated tender and enlarged lymph nodes (glandular tularemia; Table 1). Lymphadenopathy was located in the lower extremities in 156 (62.7%), the upper extremities (axilla or chest) in 39 (15.7%), the head/neck region in 41 (16.5%), and in an unspecified location in 13 (5.2%) cases. A single case of oculoglandular tularemia was reported. Eight participants reported a transient pharyngitis, but symptoms were otherwise not consistent with oropharyngeal tularemia. Twenty-four (7.3%) participants had persistent fever without localized symptoms, suggestive of typhoidal tularemia. Other symptoms included diarrhea and/or vomiting (n = 17; 5.2%) and headache (n = 84; 25.7%). Onset of primary lymphadenopathy and/or skin lesion and fever generally occurred within the same time frame (median, 0 days; IQR, −2 to +1 days).

Respiratory symptoms (cough, shortness of breath, pleuritic chest pain) were described among 59 (18.0%) participants. Among these who had chest radiograph imaging, 25/40 (62.5%) had confirmed infiltrates and 5/40 (12.5%) cases had atypical imaging findings. Fifteen cases had confirmed infiltrates on computed tomography (CT) scans, with 14 scans reporting nodular infiltrates (5 involving multiple lobes), 4 suspected pulmonary abscesses, 8 enlarged thoracic lymph nodes >10 mm, and 4 necrotic lymph nodes. Six CT reports were highly suspicious for malignancy, with 3 undergoing positron emission tomography/CT scanning, and 1 CT-guided lung biopsy. Fourteen (93.3%) cases underwent repeat CT of the chest with partial or complete resolution of infiltrates/lymph nodes. No malignancy was identified during follow-up.

Secondary skin manifestations separate from the primary ulcer site and lymph node were self-reported among 44 (13.5%) participants. Rashes were documented in 34 (10.4%) cases, with characteristics reported in 16 cases. Of these, 2 were erythema nodosum, 6 vesicular, 2 pustular, 4 macular, and 2 papular. Sites of secondary rashes were lower limbs (n = 16; 61.9%), upper extremities (n = 12; 57.1%), trunk and/or back (n = 7; 33.3%), and/or head and/or neck (n = 6; 28.6%). Palmar involvement was described in a single case, whereas no mucosal involvement was reported. Median time until onset of rash was 10 days from symptom onset (n = 11; range, 4–20 days). In 19 cases, rashes occurred after initiation of antibiotic treatment (median, 3; range, 0–5 days), whereas 4 developed a rash before starting treatment.

Participants waited a median of 4 days (IQR, 3–7 days) from symptom onset until seeking healthcare. First point of contact were GPs (n = 229; 70.0%), after-hours GPs (n = 48; 14.7%), emergency departments (n = 40; 12.2%), or specialist clinics (n = 9; 2.75%); 227 (69.4%) had subsequent follow-up. Fifty-two (15.9%) participants required hospital admission, with indications including suspected severe illness (n = 16; 30.8%) and/or investigation of unknown fever (n = 43; 82.7%). Admitting teams included internal/respiratory medicine (n = 26; 50%), infectious diseases (n = 19; 36.5%), pediatrics (n = 2; 3.8%), and otorhinology (n = 3; 5.8%).

An initial alternative presumptive diagnosis was frequent at presentation (n = 158; 48.3%), including unspecified viral infection (n = 51; 13.1%), bacterial skin/soft-tissue infection (n = 31; 9.5%), bacterial respiratory infection (n = 16; 4.9%), Puumala virus infection (n = 11; 3.4%), undifferentiated fever/sepsis (n = 13; 3.4%), urinary tract infection (n = 9; 2.8%), other specified infections (8; 1.8%), meningitis (n = 5; 1.5%), eczema/urticaria (n = 3; 0.9%), thromboembolic events (n = 3; 0.9%), or acute surgical pathology (n = 2; 0.6%).

**Figure 3.**
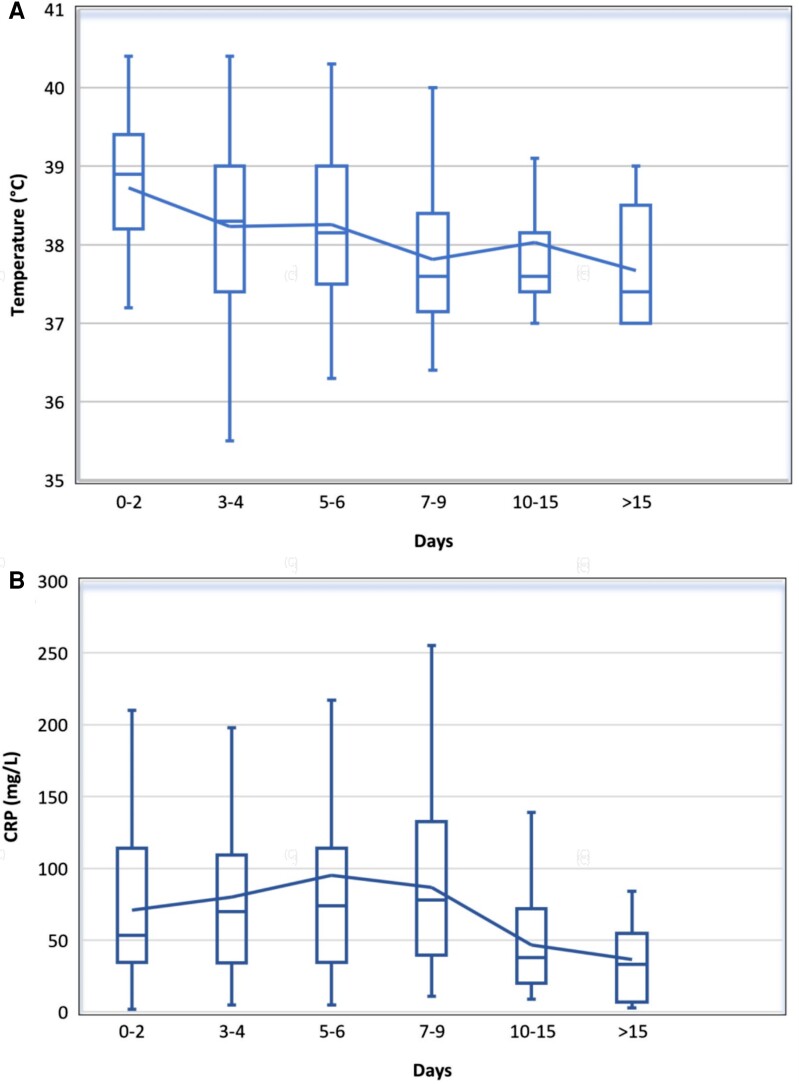
Initial recorded (*A*) temperature (°C) and (*B*) CRP and at time from onset of tularemia symptoms (n = 289). Box diagram denotes median, interquartile range, range (extreme values not included). Line denotes mean values. CRP, C-reactive protein.

Of 230 participants with temperature recorded at time of the first healthcare visit, 137 (59.6%) had a temperature ≥38.0°C. Median temperature was 38.3 °C (IQR, 37.5–39.0). Participants presenting later had a lower median initial temperature (Figure 3*A*). Median self-reported duration of fever was 5 days (IQR, 4–10).

### Laboratory Manifestations

Laboratory data (Table 1) were available for most patients because of capillary point-of-care testing in all primary care facilities. Among 289 (88.4%) participants who had an initial C-reactive protein (CRP) recorded, median CRP was highest on days 7 through 9 after symptom onset (78 mg/L; IQR, 40–133), and lower among participants who presented later, suggesting a spontaneous reduction over time and/or milder illness (Figure 3*B*).

Leukocyte counts remained within normal limits for most participants (≤8.8 × 10^6^ cells/mL; n = 140; 70.7%), whereas monocytosis (>0.8 × 10^6^ cells/mL) was present among 36 cases (48.0%). Procalcitonin was mildly elevated (>0.1 ng/mL) among most cases (n = 15; 88.2%), whereas few had levels above 0.25 ng/mL (n = 6; 35.3%).

### Treatment Outcomes

A total of 321 (98.2%) participants received antibiotic treatment, which was started a median of 5 days after onset of symptoms (IQR, 3–9 days). Median time until appropriate antibiotic regimen (defined as a quinolone, tetracycline, or aminoglycoside) was 7 days (IQR, 4–14 days).

**Figure 4.**
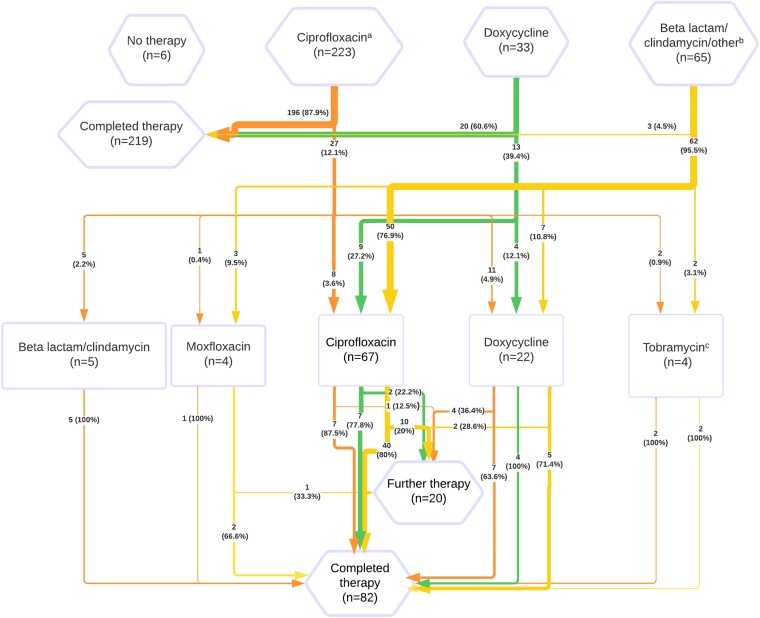
Antibiotic treatment (and retreatment) pathways for laboratory-confirmed tularemia cases (n = 327). Rectangles represents retreatment. ^a^Including 2 cases combined with 5 d of tobramycin (not requiring further retreatment). ^b^Other treatments included: rifampicin/isoniazid/pyrazinamide/ethambutol for tuberculosis regimen (n = 1), and erythromycin (n = 1). ^c^In combination with vancomycin (n = 1), ciprofloxacin (n = 2).

Initial antibiotic treatment (Figure 4) was most often ciprofloxacin (n = 223; 68.2%). Twenty-five participants (7.6%) had a previous documented allergy to antibiotics, predominantly beta-lactams (n = 20). Overall, 71 (21.7%) participants did not receive an appropriate first-line antibiotic, of which 6 (1.8%) received no documented antibiotic treatment.

### Retreatment

A second antibiotic agent was given to 102 (31.2%) of participants. Of these, 62 (60.7%) had received an inappropriate first-line antibiotic (Figure 4).

Among participants initially treated with ciprofloxacin, 27 (12.1%) received retreatment. Of these, 7 (25.9%) had <10 days of initial treatment. Reported causes for retreatment were suspected bacterial superinfection (n = 2), adverse drug reaction (n = 8), or suspected treatment failure (n = 17; nonmutually exclusive: persistent lymphadenitis [n = 9], persistent/recurrent fevers [n = 3], persistent arthralgia/suspected reactive arthritis [n = 2], persistent cough [n = 2], peri-/postinfectious lethargy [n = 2], relapsing myopericarditis [n = 1]).

Among participants receiving initial treatment with doxycycline, 13 (39.4%) were retreated. Four (30.8%) had received subtherapeutic doses, and 10 (76.9%) were medicated for <14 days. Among those successfully treated with doxycycline, only 1 (5%) had subtherapeutic dosing, whereas 5 (25%) had treatment <14 days. Reported causes for retreatment were optimization of antibiotics after tularemia diagnosis (n = 2), adverse drug reaction (n = 2), and suspected treatment failure (n = 9; peri-/postinfectious lethargy [n = 4], persistent lymphadenitis [n = 4]; persistent/recurrent fevers [n = 3], persistent cough [n = 3]).

Persistent lymphadenopathy (after 1 course of appropriate antibiotics) was present among 22 cases (6.7%); with 6 (27.3%) having spontaneously draining suppurative lymph nodes, 6 (27.3%) receiving fine-needle aspiration, and 4 (18.2%) incision and drainage. Three aspirates were *F. tularensis* PCR positive and 1 was culture positive; however, not all aspirates were sent for analysis.

### Multivariable Analysis of Treatment Outcomes

Participants who experienced treatment failure after receiving an appropriate antibiotic regimen were compared with those who had successful treatment (Table 2). Variables were analyzed using multivariable analysis after correction for multiple comparisons and included time until receiving appropriate antibiotics (8 [IQR, 3.25–20.75] vs 7 [IQR, 4–11.25] days; adjusted *P* = .076), doxycycline-based treatment regimen (vs ciprofloxacin; adjusted *P* = .084), and participant age (years; adjusted *P* = .270). Thus, no significant correlations were identified.

**Table 2.** Comparison of Characteristics of Participants With Completed Treatment After Appropriate Antibiotic Regimens vs Those Requiring Further Treatment Courses

	Completed Treatment After Appropriate Antibiotic Regimen (n = 267)^a^	Retreatment After Appropriate Antibiotic Regimen (n = 45)^b^	Missing Values	Univariate *P* Value	Multivariable *P* Value (adjusted)
Gender				.506	
Male	133 (49.8%)	25 (56.5%)			
Female	134 (50.2%)	20 (43.5%)			
Age (y)	54 (IQR, 40–65)	57 (IQR, 47–66)		.156	.270 (.270)
CRP (mg/L)	55 (IQR, 30–102)	67 (29.5–120.5)	37 (11.9%)	.650	
Leukocytes (×10^6^ cells/mL)	7.50 (IQR, 6.20–9.05)	7.60 (IQR, 6.25–9.38)	104 (33.3%)	.833	
Days until seeking healthcare	4 (IQR, 3–7)	3 (IQR, 2–5.75)	13 (4.2%)	.045	
Days until appropriate antibiotic	7 (IQR, 4–11.25)	8 (IQR, 3.25–20.75)	13 (4.2%)	.210	.026 (0.076)
Respiratory symptoms	47 (17.5%)	8 (17.8%)		.928	
Year of treatment				.518	
Nonepidemic year	43 (16.1%)	9 (20.0%)			
Epidemic year (2012, 2015, 2019)	224 (83.9%)	36 (80.0%)			
Initial appropriate regimen				.024	.043 (.084)
Ciprofloxacin	240 (89.9%)	35 (77.8%)			
Doxycycline	27 (10.1%)	10 (22.2%)			
Antibiotic regimen with appropriate dose and duration			18 (5.8%)	.500	[.524]^c^
Ciprofloxacin ≥500 mg twice daily ≥10 d	239 (89.5%)	27 (60.0%)			
Doxycycline ≥100 mg twice daily ≥14 d	24 (9.0%)	4 (8.8%)			
Immunosuppression	7 (2.6%)	1 (2.2%)		.876	

Logistic regression for categorical variables. Mann-Whitney *U* test for ordinal and continuous variables. Data reported in absolute numbers and percentages (in brackets), or median and interquartile range, as specified. Significance in multivariable analysis after Holm-Šídák correction applied for adjusted *P* values is displayed in brackets.

Abbreviations: CRP, C-reactive protein; IQR, interquartile range.

^a^Defined as regimen containing ciprofloxacin, aminoglycoside, or doxycycline, as per the World Health Organization.

^b^Retreatment despite a course of ciprofloxacin or doxycycline, individuals with “optimization” of antibiotics after diagnosis (n = 2) or adverse drug reactions requiring treatment with alternative regimen (n = 11) were excluded.

^c^Value included as alternative to “initial appropriate regimen” in multivariable analysis and not part of formal multivariable model.

## DISCUSSION

In this retrospective cohort study of 327 tularemia cases in northern Sweden, we demonstrate that tularemia is a multifaceted disease with a multitude of differential diagnoses and wide range of clinical manifestations, including prolonged suppurative lymphadenitis and atypical respiratory infection. We further describe successful treatment outcomes among individuals receiving early treatment with ciprofloxacin and doxycycline and highlight time until appropriate treatment as a potential cause for requiring retreatment, as previously noted [27].

Our cohort had similar demographics to previous studies performed in northern Europe [3, 9, 11, 28–31] and identified men and women aged 40 to 60 years as having the highest burden of tularemia, although there was high prevalence of infection throughout all age groups. Our data support ulceroglandular tularemia transmitted by mosquitos as the predominant clinical form in this geographical region [6, 31], pulmonary tularemia as an important cause of pneumonia, and atypical disease often misdiagnosed as malignancy and/or tuberculosis [9, 32]. Immunosuppression was infrequent in our cohort (n = 8), with 4 cases admitted to the hospital, including 1 developing pulmonary tularemia and 2 cases with *F. tularensis* septicemia.

Our findings show secondary skin manifestations in approximately 15% of cases, consistent with previous studies [9, 33–35]. Children aged 10 to 15 years appeared to have frequent skin involvement (54.5%), as previously described [36]. Interestingly, secondary skin manifestations appeared in close relation to commencing appropriate antibiotics, and one could speculate about a correlation between the 2. We highlight that limited knowledge about these reactions can lead to potentially inappropriate allergy labeling, as evidenced by our cohort.

Reported laboratory values in our cohort identified CRP (50–150 mg/L in 45.3%), procalcitonin (>0.1 ng/mL in 88.2%; <0.5 ng/mL in 94.1%) and monocytes (>0.8 × 10^6^ cells/mL in 48.0%) as nonspecific markers for tularemia infection in the context of high clinical suspicion [16]. Previous studies have predominantly reported normal differential counts in type B tularemia, with monocytosis >0.95 × 10^6^ cells/mL present in 4% of cases compared with 30.6% in our cohort [9, 37]. Leukocyte differential count is not routinely performed in primary care settings in Sweden. Perhaps wider implementation could help guide diagnosis of atypical manifestations of tularemia.

Chronic suppurative lymphadenitis has previously been described in up to 30% of patients with ulceroglandular and glandular tularemia [1, 9]. We describe chronic suppuration in 8.8% of cases, often leading to surgical intervention and/or retreatment with multiple courses of antibiotics. Interestingly, among PCR-positive cases, culture negativity was associated with longer time since symptom onset (median, 10.5 days; IQR, 6.5–23.0 vs 6 days; IQR, 4.75–8.5; *P* = .029). It remains unclear whether replicating bacteria are present in the ulcer after completed treatment or whether persistent lymphadenitis is primarily driven by an inflammatory response, as highlighted by few PCR-positive cases after aspiration and only a single culture positive [38]. Rates of incision and drainage appear to be lower in our cohort than in studies from France (27%) and the United States (19%) [13, 39], perhaps potentially related to shorter time until institution of appropriate antibiotic treatment. Even higher rates (60%) have been reported in pediatric populations [40]. The clinical value of prolonged antibiotic treatment in drained suppurative lymphadenitis remains unclear and will require further studies, potentially stratified by PCR status and/or culture.

Our cohort appears to have lower retreatment rates after appropriate antibiotic regimens than previous studies (16.8% vs 38.6%) [13]. This could be explained by the short duration to receiving appropriate therapy, as observed in another Swedish study with 95.3% successful treatment rate after initiating therapy early (median, 3 days from symptom onset) [19]. We further show successful treatment in 3 of 4 (75%) of cases treated with moxifloxacin as a first- or second-line antitularemia agent, as supported by previous in vitro studies [17]. As previously described [6, 17], treatment with beta-lactams or clindamycin appears ineffective.

Comparisons between ciprofloxacin and doxycycline for treatment of tularemia have previously been limited by sample size [13, 18, 20, 22, 39]. In our cohort, we show a multivariable trend toward requiring retreatment in those receiving doxycycline compared with ciprofloxacin (adjusted *P* = .076); however, when accounting for appropriate dosing and duration of antibiotic therapy, there was no significant difference between groups (14.2% vs 10.2%; *P* = .524). Although this is consistent with previous studies showing that shorter treatment periods are associated with relapse [22], our study is likely underpowered for assessment of this outcome.

Interestingly, we observed a broad range of differential diagnoses among clinicians, leading to 19.9% of participants receiving empiric antibiotics ineffective against tularemia. Higher rates were observed during nonepidemic years, suggesting that improved awareness and high clinical suspicion is required among clinicians to promote overall outcomes given that initial serological results can be negative [9].

No deaths were recorded in our cohort, and only 3 deaths were reported to the Swedish Cause of Death Register between 1997 and 2022, suggesting that, although type B tularemia can cause serious morbidity, it is rarely fatal.

Our study was limited by its retrospective design. Despite being 1 of the largest descriptive studies of clinical outcomes in tularemia, the sample size limited statistical analysis. Antibiotic treatment outcomes are difficult to interpret in those receiving retreatment because cases often received overlapping antibiotic regimens. The addition of a questionnaire to study participants improved the quality of clinical data by assessing long-term outcomes, but because of the long time between infection and time of survey in some cases, recall bias and limited memory could have influenced these results. Respondents were slightly older and more likely to be female compared with overall reported cases, introducing a selection bias that could have skewed the results. While our results should be generally applicable to type B tularemia in northern Europe, the level of relevance remains unclear in settings where alternative clinical and microbiological forms dominate.

## CONCLUSION

In conclusion, we comprehensively summarize clinical, laboratory, and treatment outcomes of type B tularemia. Targeting tularemia remains challenging because of its multifaceted presentation with a wide range of differential diagnosis and unclear optimal treatment regimens. We believe the present study can provide guidance regarding targeted treatment and highlight areas for future research. Ideally, a randomized controlled trial to evaluate effectiveness of antibiotic therapies, as well as the possible need for prolonged antibiotic treatment in suppurative lymphadenitis, should be conducted to improve clinical outcomes.

## Supplementary Data

Supplementary materials are available at *Clinical Infectious Diseases* online. Consisting of data provided by the authors to benefit the reader, the posted materials are not copyedited and are the sole responsibility of the authors, so questions or comments should be addressed to the corresponding author.

## Supplementary Material

ciae098_Supplementary_Data

## Notes

***Author contributions***. Conceptualization: T. N. G., M. P., A. S., R. L., and A. N. Investigation: M. P., T. N. G., and R. L. Formal analysis: M. P., T. N. G., and R. L. Writing – Original Draft: M. P. and T. N. G. Writing – Review & Editing: T. N. G., A. S., M. P., A. N., and R. L. Supervision: T. N. G.

***Acknowledgments***. The authors thank the study participants for their valuable contributions.

***Data Availability***. Data not publicly available.

***Financial support***. This work was supported by generous funding from the County Council of Norrbotten (NLL-933177 to T. N. G.); Umeå University through a regional agreement with the County Council of Norrbotten (ALF Universitets-ST to T. N. G.); and from County Council of Västerbotten (RV-966950 and RV-939171 to A. S.).
