# Correction to: Targeting Tularemia: Clinical, Laboratory, and Treatment Outcomes From an 11-year Retrospective Observational Cohort in Northern Sweden

**DOI:** 10.1093/cid/ciae175

**Published:** 2024-04-30

An error appeared in the corrected proof publication of this article (Plymoth et al. “Targeting Tularemia: Clinical, Laboratory, and Treatment Outcomes From an 11-year Retrospective Observational Cohort in Northern Sweden.” *Clin Infect Dis*. https://doi.org/10.1093/cid/ciae098). The second author was incorrectly listed as Robert Lundquist. The article has been corrected to list the second author as Robert Lundqvist.

