# Supplementary material for: Targeting Tularemia: Clinical, Laboratory, and Treatment Outcomes From an 11-year Retrospective Observational Cohort in Northern Sweden
Source: Clin Infect Dis. 2024 Feb 23;78(5):1222–31. doi: 10.1093/cid/ciae098 (PMC11093680; doi:10.1093/cid/ciae098)
Supplement: ciae098_Supplementary_Data [file ciae098_supplementary_data.docx]

**Supplement**

**Multivariable regression model**

1. Unmatched univariate analysis was performed on continuous (Mann-Whitney U test) and categorical variables (logistic regression). Variables with p<0.25 were added to a multivariable logistic regression model.
2. Variables showing multicollinearity (variance inflation factor [VIF]>5 in linear logistic regression including independent dummy categorical and continuous variables) or for which the proportion of missing data was >10%, as well as those that only concerned a minor subgroup of participants were excluded; this corresponded to the following variables: CRP; leukocyte count; days with fever; antibiotic regimen with appropriate dosing (this was run as a parallel multivariable model, replacing **Initial appropriate regimen)**
3. The model was adapted through a stepwise backwards elimination protocol as cited in the methods section (table 1). Variables with p<0.10 were kept in the model. as well as variables for which elimination led to >15% confounding effect on remaining variables.
4. All eliminated variables were then re-entered, and those with p<0.15 were kept in the model. The final model is presented in the manuscript.

**Table 1. Steps in backwards elimination protocol for multivariable regression model.**

| Step | | Regression coefficient of constant | Removed variable | | p-value removed variable | Goodness of fit (H&L) | Change in coefficient of constant | | | Comment |  |
| --- | --- | --- | --- | --- | --- | --- | --- | --- | --- | --- | --- |
| 1 | | -2.717 | | Days until seeking healthcare | 0.667 | | 0.494 | |  | Removed | |
| 2 | | -2.776 | | Age | 0.322 | | 0.964 | | 1.02175 |  | |
| 3 | | -2.259 | |  |  | | 0.610 | | **0.81376** | **Age status kept in equation** | |
| 4 | | -2.776 | | Pulmonary tularemia |  | | 0.964 | | 1.02175 | Removed | |
| 5 | | -2.808 | |  | 0.342 | | 0.928 | | 1.01152 |  | |
|  | |  | | **Added variable** | **p-value added variable** | |  | |  |  | |
| 6 | |  | | Days until seeking healthcare | 0.675 | |  | |  | Not added | |
| 7 | |  | | Pulmonary tularemia | 0.173 | |  | |  | Not added | |

(8) Antibiotic regimen with appropriate dosing was entered into the model replacing initial appropriate regimen**.** Other variables were not adjusted. P=0.276 (unadjusted), adjusted p-value=0.524**.**
